# Study protocol for a mixed-design evaluation of self-assured parents – A parenting support program for immigrant parents living in deprived areas in Sweden with teenage children

**DOI:** 10.1016/j.puhip.2022.100270

**Published:** 2022-05-04

**Authors:** Therése Skoog, Sabina Kapetanovic, Emma Sorbring

**Affiliations:** a Department of Psychology, University of Gothenburg, PO Box 500, 405 30, Göteborg, Sweden; b Department of Social and Behavioral Studies, University West, 461 86, Trollhättan, Sweden

**Keywords:** Immigrant parents, Parenting program, Parental efficacy, Adolescent children

## Abstract

**Background:**

Immigrant parents of adolescents experience challenges in their role as parents in the new country and express a need for parental support. Still, they are underrepresented in existing parenting programs and when they do attend, their parenting practices improve less than what they do among native parents. Self-assured parents (SAP; *Swe*. Trygga Föräldrar) targets immigrant parents living in deprived areas in Sweden who worry about their adolescents' adjustment. This study's purposes are to examine if SAP is a feasible intervention in Swedish municipalities and if SAP is effective in reaching its aims, namely to promote parental self-efficacy and parent-adolescent communication and to reduce parents' worries in the target group.

**Methods:**

SAP will be evaluated when implemented by social workers in three Swedish municipalities using a culturally-informed mixed design procedure. Parents will be recruited to the program by local social workers. Groups leaders will be interviewed, observed, and they will fill out self-reports to measure implementation quality, including fidelity and acceptability. A group of parents will be interviewed to better understand their perceived challenges and needs in their parenting in Sweden and their experience of participating in SAP. An interrupted time series design with three measurements before, two measurements during, and two measurements after the intervention has ended will be employed using self-reports of parental self-efficacy, parent-child communication, and parents' worries. Informed consent will be collected from all study participants.

**Discussion:**

Immigrant parents living in deprived areas is an understudied and marginalized population. There is a lack of culturally-informed, evidence-based parenting programs aimed at this group in Sweden. The need for specifically developed programs for immigrant parents living in deprived areas with teenage children, has been voiced by both immigrant parents themselves and the Swedish government. Thus, this study will contribute not only to the scientific literature, but also to social service practice and potentially policy making.

## 1 Background

This protocol describes a Swedish research project on the implementation and effect of a locally developed parenting program for immigrant parents with teenage children living in deprived areas who are worried about their children's well-being and adjustment. About 2% of the Swedish population live in deprived areas, where there is a high proportion of unemployed adults, low income residents, and low education levels. The majority of the population in these areas come from foreign cultures [1]. Another characteristic of these areas is their high crime rates, in which also young people are involved. In order to prevent young people from engaging in criminal, and other antisocial activities, and to promote the best possible outcomes for these children more attention and preventive programs from social services are needed.

Parental support delivered through structured parenting programs is a form of prevention that is generally effective in preventing crime and substance abuse during adolescence [2] and is part of the interventions delivered by the social services in Sweden and elsewhere [3]. Nevertheless, evidence-based methods are still lacking to support immigrant parents who live in deprived areas and whose teenage children are at particular risk of developing problem behaviors.

Parents who have recently come to Sweden often live in deprived areas and have poor orientation in Swedish society, which makes it even more difficult to take on the role of a parent and protect their children from harm compared to what is the case for native parents [4]. Empirical research shows that immigrant parents experience challenges in their role as a parent in the new country and express a need for parental support [5]. Despite this, parents with foreign background are underrepresented in existing parenting support programs [6]. Parenting support programs used among immigrant parents show poor parent participation and weaker effects on parenting practices [7]. Parenting programs aimed at immigrant parents are customized to Swedish or general international (US) conditions, which may be one of the reasons for unsatisfactory participation and results. Such programs have received critique of being stigmatizing because of their emphasis on middle-class family values [8]. The universal evidence-based programs could thus be less relevant (i.e, not ecologically valid) in groups of parents with other backgrounds than that of the majority culture. Offering culturally informed interventions may be more efficient [9]. A recent meta-analysis revealed that parenting programs with deep structure sensitivity (e.g., by addressing cultural and contextual influences on parenting), had the highest impact on positive parenting behaviors in ethnic minority groups [10]. Thus, if we are to support immigrant parents adequately and reduce the risk of the child's negative development, there are reasons to think that is should be in the form of parenting support specifically aimed at this group of parents. However, instead of authorities formulating parents' needs, the emphasis should be put on forming democratic practices with goal of supporting parents' empowerment and self-confidence on parents' own terms [3].

### 1.1 Theoretical framework of Self-Assured Parenting

**Fig. 1:**
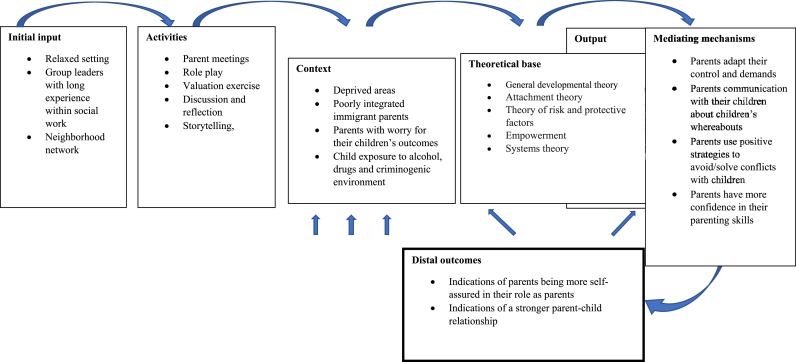
Logic Model of Self-Assured Parenting program.

The SAP program can be defined as a selective prevention program [11] as it targets the subgroup of immigrant parents living in deprived areas who worry about their adolescent children's adjustment. The main goal is to strengthen protective factors (e.g., parent-adolescent communication and parental self-efficacy). A secondary goal is to reduce risk factors (e.g., uninvolved parenting). Originally, the program theory behind the program was not explicitly stated in the manual. The program theory has been reconstructed by the first and second authors of this study protocol [12]. Fig. 1 presents the reconstructed program theory/logic model behind the SAP program. During the reconstruction process, which was conducted based on the recommendations by Rossi and colleagues [13], it was discovered that the program rests on a solid, theoretical foundation. General developmental theory, attachment theory, theories of risk and protection, systems theory, and theories of empowerment are main theoretical frameworks. Finally, the general approach of the program is based on the principles of the UN Convention on the rights of the child.

### 1.2 Research objectives

The purpose of the project is to test and evaluate the SAP program in deprived areas in Swedish municipalities with immigrant parents who are worried about their teenage children. Specifically, the project will provide more insight into whether SAP promotes parental self-efficacy, parent-child communication and relationship quality, and reduces parental worries. Moreover, it will monitor the implementation process closely. Given the legion challenges in completing and implementation both the intervention, and the proposed research of the intervention, studying implementation is crucial. Using a mix of quantitative and a qualitative designs the following four research questions will be answered:

RQ1. What needs of parenting support do immigrant parents who live in deprived areas express?
RQ2. How do immigrant parents who live in deprived areas experience SAP?
RQ3. What are the barriers and facilitator factors in the implementation of SAP?
RQ4. Does SAP contribute to increased parental self-efficacy and communication with their teenage children and does it reduce parents' worries about children's psychosocial outcomes?

## 2 Methods/design

### 2.1 Research design

**Fig. 2:**
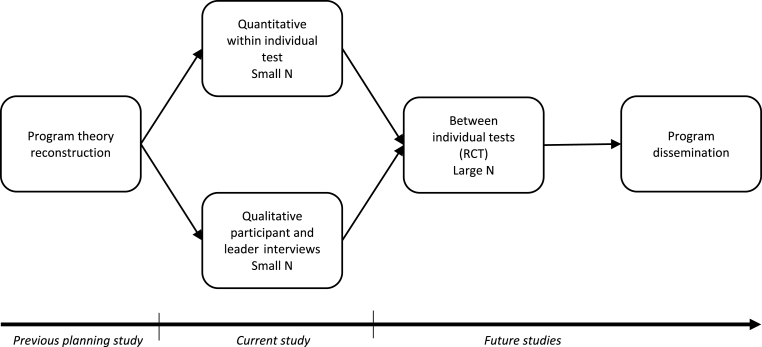
Overall study design.

This research project stems from a request from social workers to systematically evaluate their practice, i.e. the SAP program. This request from the practitioners has fundamentally affected the research design of the project. The project goal is to build scientific knowledge that is relevant and useful for practice and to design an evaluation that is feasible in the context of everyday social service practice. Such partnership between the social workers and the researchers can generate evidence about the preventive efforts used by practices [14]. Moreover, not only the intervention should be culturally competent, but the evaluation needs to be as well [15]. Therefore, we have adopted a culturally informed approach to evaluation of the SAP program. Fig. 2 presents the overall model for the evaluation. The model is based on Johnston and Smith's [16] recommendations concerning how practice-based evidence is best developed. Although a randomized controlled trial (RCT) is considered to be the golden standard when evaluating the efficacy and effectiveness of interventions the validity of RCT, at least in the first attempts to evaluate a program involving ethnic/cultural minority groups, or marginalized groups in general, has been questioned [17]. We want to proceed with caution and have evidence that SAP is acceptable to social workers and parents, and effective in achieving the primary outcomes in a smaller group of participants, before delivering SAP to larger groups of parents and conducting an RCT. In the exploratory phase, where the evaluation process of SAP currently is, other designs, single-subject oriented designs in particular, have been recommended as a first choice [17]. The first phase of the evaluation of SAP was carried out during the preparatory year funded by the Public Health Agency of Sweden in 2019. During that phase, we identified the program theory and logical model behind SAP and evaluated its theoretical potential to achieve the desired outcomes. We concluded that the SAP program was a theoretically promising intervention. The current study protocol concerns the next evaluation phase 2021–2023, with a focus on "quantitative single case evaluation" and "interviews of participants and group leaders." The following stages, "randomized control trial" and "program dissemination" are not part of this protocol. Establishing high implementation quality is an important first step in evaluating interventions. If the implementation quality is low, an otherwise effective intervention will probably not have any beneficial effects. Based on previous research on implementation studies [18], we will monitor the implementation process closely through observations and leaders', mangers' and participants' “consumer satisfaction” reports.

### 2.2 Participants

The research project will be conducted in Sweden. The project has two target groups. The primary target group is parents living in deprived areas in different municipalities. The social services in the different municipalities will recruit parents to the intervention. The target parent group is parents of teenagers (children aged 12–18 years) who express worries that their teenage children will end up in risk environments, with substance abuse and crime. The parents will take part in the SAP program to increase their personal resources and foster parent-child communication to more adequately protect their children from negative developmental outcomes (i.e. substance abuse and delinquency). Parents of younger children and parents who are already participating in other parental support programs will be excluded. Because the availability of participants targeted by the SAP program is limited, the goal is an N of 30 parents that can be followed over time. In singles-subject oriented designs, a sample of *N* = 30 is considered feasible for analyses [19]. An oversampling of 30 individuals is calculated to adjust for non-consents and dropouts. Social workers who will deliver the SAP program in their municipalities constitute the second target group. All group leaders who delivered the SAP program (at least 2 in each municipality) and their managers will be asked to partake in interviews, fill in self-reports, and be observed by the program developers within the context of the current study.

### 2.3 Procedure

#### 2.3.1 Implementation study (RQ1-RQ3)

One part of the implementation evaluation will concern parents' perceived challenges and needs in their parenting in Sweden, answering RQ1. The second part will concern parents' experience of participating in the SAP program answering RQ2. Interviews with participants will be conducted in conjunction with the measurements at -1 week (i.e. one week before the start of the intervention) and 14 weeks (i.e. 1 month) after the end of the intervention. All participants in the effect study (see below) will be asked to participate in the interviews. Although guidelines of standards for sample size in qualitative research are generally lacking, our goal is for at least 10 parents to participate. These individual interviews of a structured nature will focus on the questions regarding parents' challenges and needs in parenting and their expectations and experiences of their participation in the SAP program. To answer RQ3, we will conduct interviews with group leaders and managers per delivered course. In addition, leaders will fill out self-reports. Proctor et al. [20] identified eight specific implementation factors central for the outcomes of the implementation, including acceptability (e.g. perceived satisfaction in stakeholders), feasibility (the extent to which the intervention can be carried out by practitioners) and fidelity (the extent to which an intervention is delivered as intended). The interview guides are presented in Table 1. All implementation factors will be evaluated by group leaders and/or their managers' self-reports after each completed intervention. The self-reports have been designed to measure the implementation factors according to Proctor et al. [20]. Table 1 presents a detailed description of the outcomes to be examined.

**Table 1:** Overview of the implementation factors in the interview guide.

Implementation factors	Description of the factor	Example	Scale anchors
**Structural aspects**			
Acceptance	The extent to which the leader acknowledges the method	SAP program is appealing to me	1 – not at all 5 - strongly agree
Adoption	The extent to which the leader wants to take in the program	SAP program has my permission to be used in our organization	1 – not at all 5 - strongly agree
Appropriateness	The extent to which the leader perceives the program to be appropriate for the target group	SAP program is appropriate for the target group	1 – not at all 5 - strongly agree
Cost	The extent to which the leader perceives the program to be cost efficient	It pays off to work with SAP program	1 – not at all 5 - strongly agree
Feasibility	The extent to which the leader perceives the program to be practical	SAP program curriculum is easy to use	1 – not at all 5 - strongly agree
Penetration	The extent to which the leader perceives the program to be integrated within their agency	It is possible to implement the SAP program in our organization	1 – not at all 5 - strongly agree
Fidelity	The extent to which the intervention is delivered as intended	Did you work with the program as described in the manual?	1 – Yes, completely 5 - No, not at all
Dosage	The amount of intervention provided to the target group	On average, how much intervention did the parents receive?	1 – completely (100%) 5 – none (0%)
**Responsiveness**			
Enthusiasm	The extent to whether the leader perceives the parents/themselves to be enthusiastic during the intervention	How enthusiastic were the parents/you during the intervention?	1 – not at all 5 – completely
Engagement	The extent to whether the leader perceives the parents/themselves to be engaged during the intervention	How engaged were the parents/you during a typical session?	1 – not at all 5 – completely
Knowledge/skills	The extent to whether the leader perceives the having knowledge and skills to work with SAP	Were you confident in your skills to work with SAP program?	1 – not at all 5 – completely

#### 2.3.2 Effect (RQ4)

The effect evaluation focuses on the effect of SAP on the primary outcomes, parents' self-efficacy, parent-child communication as well as parents' worries about their children's development. It was designed to answer RQ 4 focusing on perceived change in the primary outcomes. As a feasible alternative to RCTs, single-subject designs have gained an increasingly prominent place in the literature on evaluations of social interventions aimed at different forms of problem behavior among young people, not least in subpopulations such as ethnic minority groups [21]. In this study, we will use an interrupted time series design (ITSD, 17). ITSD is usually based on a series of within-individual measurements of the outcome before, during and after exposure to the intervention. Well-executed ITSD have causal inference properties that resemble RCTs [22]. As recommended by Kratochwill et al. [23], measurements will be made on two occasions before the intervention, on three occasions during the ongoing intervention, and on two occasions after the end of the intervention. This is 7 measurements in total per participating parent. Due to the characteristics of the target group, the number of instruments used will be limited and focused on assessing the primary outcome measures. The instruments are highly structured and will be administered via online survey platform. Parents will be asked to answer in relation to the child that they are the most worried for. The questionnaires will be provided in Swedish, English, Somali, or Arabic according to parents own choice.

*Parental self-efficacy* will be measured by the Parenting Sense of Competence in Parenting Scale (PSOC; [5,24]). The scale has subscales measuring satisfaction in parenting (9 items) (e.g. “Although parenting can be rewarding, I am frustrated now that the child is the age he/she is”) and parental self-efficacy (7 items) (e.g. “I think I have what it takes to be a good parent to my child”). Answers are provided on 6-point Likert scales (1 - *fully agree* to 6 – *completely disagree*).

*Parents' worries* [25] about their children will be assessed on a scale comprising six questions (e.g. "Are you worried that your child will get caught by the police?”) answered on a 5-point Likert scale (1 - *yes, always* to 5 – *no, never*).

*Parent-child communication* [26] will be assessed using an instrument with 22 questions measuring 1) Parent knowledge of child whereabouts (6 items) (e.g. "Do you know what places the child visits when he/she is out with his friends in the evenings?") 2) Parent control (5 items) (e.g. "Does the child need permission to stay out late on a weekday evening?") 3) Parent solicitation (6 questions) (e.g. "Do you ask the child to tell you about things that happen in his/her spare time?") and Child Disclosure (5 items) (e.g. "When the child has been out one evening, does he/she want to tell you about what she/he has done?"). A 5-point Likert scale (1 - *almost never* to 5 - *very often*) will be used.

Demographic information on parents' ethnicity, age, educational level, employment, housing situation, family structure, and the number of children, children's age, class, and gender will also be collected.

### 2.4 Ethical considerations

The SAP study has been approved by the Swedish Ethical Review Authority (reference number 2020-04510; 2021-03239). We will obtain written informed consent from all participating parents and social workers before collecting any data. Parents and social workers will be informed orally and in writing about the study and the study procedures including how data will be handled and stored. We will inform parents in such a way that they can fully understand what it is that we are asking them to consent to and how we will use the data. Therefore, we will have information letters available in Swedish, English, Arabic, and Somali. Arabic and Somali are native languages of many of the parents in the deprived areas targeted by SAP. We will stress to all potential participants that participation in the research is voluntary. We will clarify to parents that their choice regarding participation is the research project does not affect their chances of partaking in the SAP program. Participants may withdraw from the study at any time without risking any negative consequences. Because this project represents the first evaluation of the SAP program, we have chosen to keep the number of participants to a minimum to minimize any potential, unforeseen iatrogenic effects of the program. We will offer participating parents an incentive corresponding to 30 EUR.

### 2.5 Analysis

To answer the first three research questions (RQ1, RQ2, and RQ3), for which we will collect qualitative data through interviews with parents and SAP group leaders, we will use thematic analyses [27]. Thematic analysis includes descriptions of different patterns, or themes, provided in the data which generates a deeper understanding of the data material. To answer RQ3, we will also perform descriptive statistics of the group leader self-reports. To answer the fourth research question, we will use an interrupted time series design (ITSD, 17). ITSD is usually based on a series of within-individual measurements of the outcome before, during and after exposure to the intervention. The appearance of these series is compared to determine if SAP (thus the interruption) has any effect on the outcome. A difference between the pre-and post-tests indicates an effect of the intervention. Various exposed individuals' series are then studied to determine if identified patterns can be replicated. The advantage of ITSD is that data in this situation can be presented in a meaningful and informative way with the help of descriptive statistics and data visualization plotted on graphs and complemented with a celeration line [28]. With the help of these graphs, individual exposed (and non-exposed) time series can be presented and analyzed. If the celeration line differs between the different phases, changes in outcomes could be present. In addition, the conservative dual-criteria (CDC) method could be used with small data streams (<7). Using the binomial formula, the CDC method helps to judge whether the change in the variables was present relative to the baseline condition. Finally, Dallery et al.'s [29] recommendations will be followed to analyze visual representations of data.

## 3 Discussion

Immigrant parents living in deprived areas is an understudied and marginalized population. There is a lack of culturally-informed, evidence-based parenting programs aimed at this group in Sweden and elsewhere. The need for specifically developed programs for immigrant parents living in deprived areas with teenage children, has not least been voiced by both immigrant parents themselves [6]. A central idea of the described project is to evaluate a culturally sensitive and competent intervention to these parents that often struggle with the acculturation processes as well with the exposure to harmful environments. Thus, this study will contribute not only to the scientific literature, but also to social service practice and potentially policy making as successful implementation builds on an understanding of the needs and characteristics of the target group [30]. In order to develop adequate support and preventive efforts for the target families, cultural competence in the work done by the social workers as well as the researchers is critical. Without achieving culturally relevant practices and knowledge, the immigrant parents would not obtain the support based on their specific needs. As a result, they would have less possibilities to support their children. The SAP program targets an understudied population with possible cultural and language barriers, which may have an impact on the implementation of the program. Accordingly, this project does not only contain a culturally informed intervention, but also a culturally informed research design.

While the proposed research carries many advantages and has the potential to advance the field, it also has possible limitations and caveats. The proposed type of research comes with legion challenges that might threaten the validity of the research findings and even the feasibility of the project. One challenge involves reaching parents and recruiting them to the project. We believe that our close collaboration with local and culturally competent social workers will be important to tackle this challenge. Furthermore, the issue of ethnocentrism needs to be acknowledged [31]. One of the goals behind the SAP program is to help parents adjust to parenthood in Sweden and to obtain Swedish values in relation to parenthood (e.g., that open and bidirectional communication is a cornerstone of any good parent-teenager relationship). In trying to achieve this, it is important to be respectful in relation to what other cultural values parents may have. Another challenge relates to methodology and potential threats to validity. Among other things, history effects could affect the sustainability of the findings across time. Future studies are needed to handle this challenge. Further, the research design demands a lot from participants, including that they can read and that they have the motivation and possibility to fill out repeated measurements. It is possible that this will cause study attrition and exclude potential participants from taking part in the study. A final note has to do with the external validity of the findings. Future research is needed to test whether any detected effects are sustained across countries.

## Ethical approval and consent to participate

Ethical approval for this study was provided by the Swedish Ethical Review Authority (reference numbers 2020-04510; 2021-03239). Active, informed, and written (on paper or electronically) consent to participate is solicited from the participating parents and social workers before data collection commence. The consent procedure has been approved by the ethics authority.

## Funding

The research project has been funded by the 10.13039/501100004359Swedish Research Council for Health, Working Life, and Welfare (reference number 2020-01349). The funding body has no role in the design of the study and collection, analysis, and interpretation of data and in writing the manuscript.

## Trial registration

ClinicalTrials.gov #2020-01349. Registered 19 June 2020.

## Declaration of competing interests

The authors declare that they have no competing interests.
